# Case report: cerebral sinus vein thrombosis in two patients with AstraZeneca SARS-CoV-2 vaccination

**DOI:** 10.1007/s00415-021-10731-2

**Published:** 2021-10-05

**Authors:** Mathias Fousse, David Schub, Fatma Merzou, Klaus Fassbender, Martina Sester, Michael Kettner, Piergiorgio Lochner, Tina Schmidt, João Reinoldo Goi Júnior

**Affiliations:** 1 Department of Neurology, Saarland University Medical Center, Kirrbergerstrasse 90, 66421 Homburg, Saarland Germany; 2 Department of Transplant and Infection Immunology, Saarland University Medical Center, Homburg, Saarland Germany; 3 Department of Neuroradiology, Saarland University Medical Center, Homburg, Saarland Germany

**Keywords:** Cerebral sinus vein thrombosis, Vaccination, COVID-19, ChAdOx1, SARS-CoV-2

## Abstract

SARS-CoV-2 infection is associated with an increased rate of thromboembolic events and mortality. Different vaccines are globally used to limit the pandemic. In this report, we present the case of two young female patients with newly diagnosed cerebral sinus vein thrombosis occurring after injection of the vector-based ChAdOx1 vaccine. Both patients presented with unusual headache only. The two of them used an estrogen-containing contraception, had had a history of deep venous thrombosis, and both had MTHFR mutations. Both patients developed SARS-CoV-2 specific humoral and cellular immunity including both CD4 and CD8 T cells. This rare, but serious complication needs to be considered after vaccination of young females, even if there is no evidence of heparin-induced thrombocytopenia.

## Background

In the fight against Coronavirus Disease (COVID) 19 pandemic, vaccines such as AstraZeneca adenovirus vector-based vaccine ChAdOx1 play a major role. There is a growing body of evidence that in a small number of patients, ChAdOx1 is associated with a higher risk for developing cerebral sinus vein thrombosis (CSVT), a relatively uncommon coagulation disorder with an incidence of about 3–4 cases per 1 million population [1].

## Case description

**Fig. 1:**
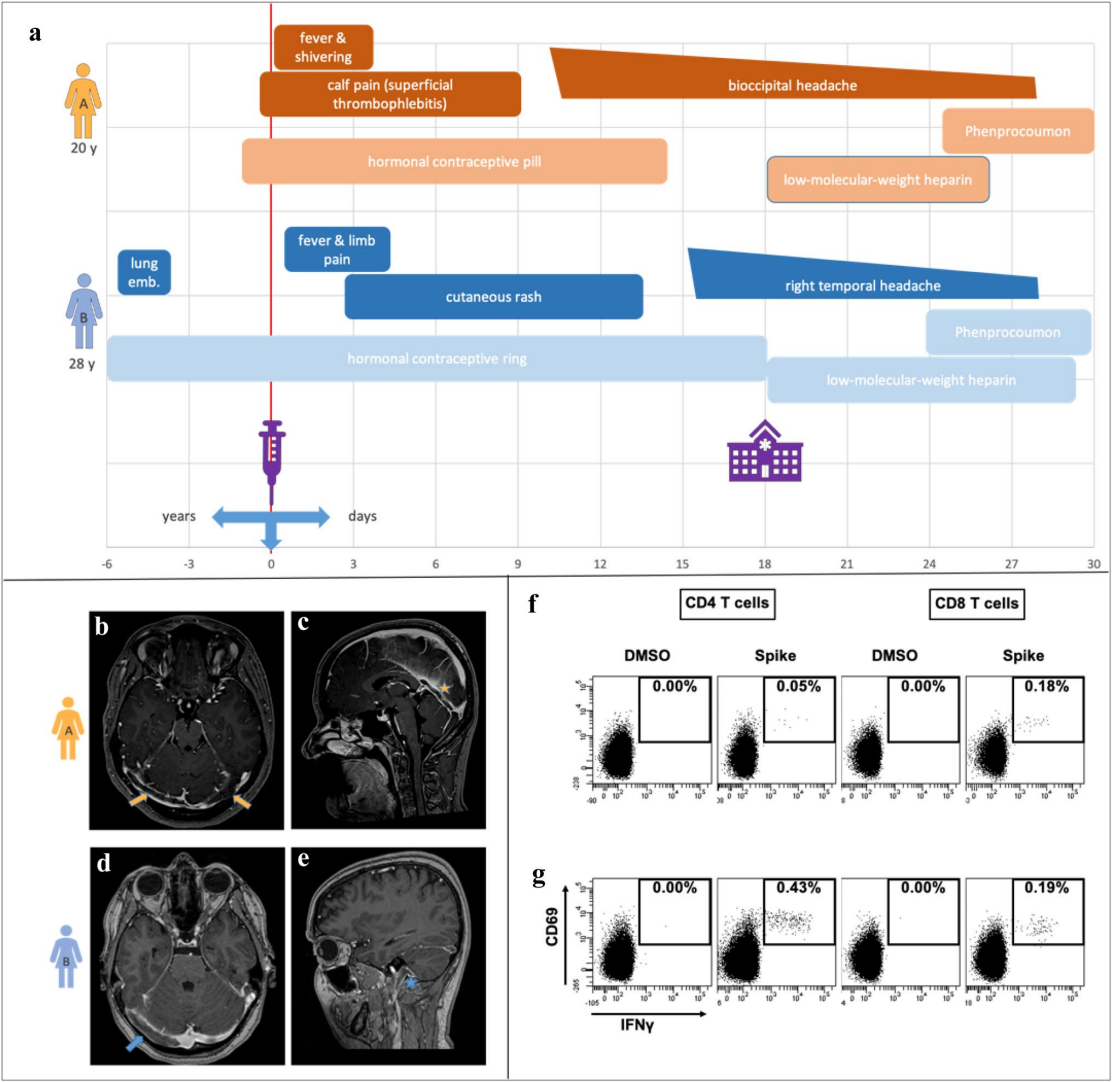
Diagnosis of sinus vein thrombosis after vaccination with ChAdOx1-S and clinical course in a 20-year-old woman (patient A) and a 28-year-old woman (patient B) (Fig. 1**a**). MRI (Fig. 1**b–e**): Axial and sagittal T1 with contrast (MP-RAGE) of patient A (Fig. 1**b** and 1**c**) with thrombosis of the transverse sinus right accentuated, right sigmoid sinus, rectus sinus, and cerebral vena magna and of patient B (Fig. 1**d** and 1**e**) with thrombosis of the right transverse sinus, right sigmoid sinus, and right proximal jugular vein. Flow cytometric analysis showing (Fig. 1**f**,  **g**) specifically activated CD4 and CD8 T cells producing IFN-g after stimulation with overlapping peptides derived from the spike protein. The upper right quadrants of the dotplots indicate the percentage of CD69/IFN-g double-positive CD4 or CD8 T cells from patient A (Fig. 1**f**) and B (Fig. 1 **g**). No reactive T cells were detected after control stimulation with dimethyl sulfoxide (DMSO). The stimulation with staphylococcal enterotoxin B (SEB) yielded a strong reactivity (data not shown)

Two female patients (A 20 years old and B 28 years old) presented to our neurology emergency department with drug-refractory headache 11 and 16 days, respectively, after receiving their first dose of ChAdOx1-S vaccine (course in Fig. 1a). There were no signs of infection, and no neurological deficits or increased intracranial pressure-related symptoms existed. Both patients used hormonal contraception (gestagen/ethinylestradiol).

Both patients had slightly elevated D-dimers [1.79 mg/l (A) and 0.83 mg/l (B)]. Magnetic resonance imaging (MRI) with axial and sagittal T1 with contrast (MP-RAGE) finally revealed a CSVT in both patient, however, without any sign of cerebral ischemic or hemorrhagic infarct (Fig. 1b–e). Patient A mentioned that she has noticed unilateral calf pain for already several weeks. Her ultrasound examination revealed a subacute thrombophlebitis of the small right saphenous vein. Patient B had a history of pulmonary embolism after patella surgery, which was followed rivaroxaban treatment for 1 year.

Laboratory testing included a screening panel for vasculitis and collagenoses as well as thrombophilia. Patient A had a slightly elevated β2-glycoprotein-IgG titer of 8.6 U/ml (normal range < 7.0 U/ml) with normal levels of cardiolipin antibodies and lupus anticoagulant. Hashimoto's thyroiditis with hypothyroidism was diagnosed [thyreoperoxidase antibodies of 121.0 IU/ml (normal range < 34.0 IU/ml)]. Finally, she also had a heterozygous MTHFR A1298C mutation, whereas patient B suffered from homozygous MTHFR C677T mutation and a heterozygous plasminogen activator inhibitor-1 gene polymorphism.

21 days after vaccination, SARS-CoV-2 IgG blood titer was 650 AU/ml in patient A and > 16,500 AU/ml in patient B. In addition, both patients had developed vaccine-specific cellular immunity, as shown by flow-cytometric analysis of the specific T-cell reactivity after whole blood stimulation with overlapping peptides from the SARS-CoV-2 spike protein [2], (Fig. 1f, g).

Estrogen-containing contraception was discontinued in both patients. Anticoagulation with low-molecular-weight heparin was started and later replaced by phenprocoumon. Both patients were discharged after 1 week without any neurological symptoms.

## Discussion

Infection with SARS-CoV-2 can lead to venous thromboembolism, ischemic stroke, and, more rarely, CSVT [3]. Few cases of CSVT, however, were now identified after vaccination the vector-based ChAdOx1 vaccine.

We identified two female patients who developed a non-septic CSVT after injection of their first dose of ChAdOx1-S vaccine. Both patients only suffered from light headache. There was no sign of cerebral hemorrhage or ischemia. Additionally, both patients had a thrombosis-related medical history and both used estrogen-containing contraceptives. These facts support the hypothesis that additional risk factors are necessary to develop vaccine-associated CSVT [1]. Genetic thrombophilia factor testing revealed an MTHFR mutation in both patients with an additional heterozygous PAI-1 gene polymorphism in patient B, but no evidence of a factor V ARG506 mutation or prothrombin G20210A mutation could be found. Gogu et al. recently demonstrated that in 114 cases of CSVT 52.6% had MTHFR gene polymorphism (45.6% homozygous mutation) for C677T or A1298C, partially associated with other evidence of thrombophilia such as hyperhomocysteinemia [4].

In association with the venous thrombosis, an elevated β2-glycoprotein-IgG blood titer might hint at an underlying antiphospholipid syndrome. Both patients developed a sufficient humoral and cellular COVID-19 antigen immune response. While SARS-CoV-2 blood antibody IgG titer in patient A was relatively low, the same titer was 25 times higher in patient B. Likewise, the proportion of reactive T cells, especially if CD4 T cells, was significantly higher in patient B (Fig. 1f, g), and similar in magnitude as in patients after SARS-CoV-2 infection [2].

Similar as for heparin-induced thrombocytopenia (HIT), the involvement of antibodies against platelets is currently discussed in the pathophysiology of CSVT cases of HIT also occurred after infection with SARS-CoV-2 [5, 6]. In case of post-vaccination HIT, German guidelines recommend IVIG therapy and refraining from administering heparin [7, 8]. There is possible evidence that CSVT with thrombocytopenia may lead to earlier symptom onset [9]. In any case, the presence of platelet factor 4/heparin antibodies is clustered in patients with CSVT (after vaccination) compared with the pre-COVID-19 period, allowing conclusions about the pathophysiology of some but not all affected patients [10]. In our cases, none of our patients had thrombocytopenia or positive HIPA assay, and both benefited from anticoagulation with heparin.

To date, there have been over 116 reported cases of CSVT after ChAdOx1vaccination in Germany. The majority of these patients were female and below the age of 65. 25 patients suffered from a lethal outcome. In a risk–benefit assessment, the European Medicines Agency (EMA) decided to add a safety update. The risk of thrombosis (just) in combination with thrombocytopenia (thrombosis with thrombocytopenia syndrome) is reported by the EMA to be up to 1 in 10,000 people [11]. Meanwhile, cases of CSVT also exist after vaccination with mRNA vaccines such as BNT162b2 [12].

Nevertheless, as CSVT was mainly observed in young women, in our opinion, the administration of this vaccine needs to be carefully assessed if a history of thrombotic events exists.

## Conclusion

CSVT should be considered even when persistent headache is the only systemic symptom after ChAdOx1vaccination, in particular in younger women. Also, the absence of thrombocytopenia does not exclude a possibly autoimmune-mediated CSVT. More evidence is required to decide if patients using hormonal contraception and/or with a history of thromboembolic events should receive a different COVID-19 vaccine.
